# Occupation and educational inequalities in laryngeal cancer: the use of a job index

**DOI:** 10.1186/1471-2458-13-1080

**Published:** 2013-11-19

**Authors:** Irene Santi, Lars Eric Kroll, Andreas Dietz, Heiko Becher, Heribert Ramroth

**Affiliations:** 1 Institute of Public Health, University of Heidelberg, Im Neuenheimer Feld 324, 69120 Heidelberg, Germany; 2 Robert Koch-Institute, Postfach 65 02 61, D-13302 Berlin, Germany; 3 Department of Otorhinolaryngology, Head and Neck Surgery, University of Leipzig, Liebigstrasse 10-14, 04103 Leipzig, Germany

**Keywords:** Laryngeal cancer, Education, Occupational indices, Exposure substance check list

## Abstract

**Background:**

Previous studies tried to assess the association between socioeconomic status and laryngeal cancer. Alcohol and tobacco consumption explain already a large part of the social inequalities. Occupational exposures might explain a part of the remaining but the components and pathways of the socioeconomic contribution have yet to be fully disentangled. The aim of this study was to evaluate the role of occupation using different occupational indices, differentiating between physical, psycho-social and toxic exposures and trying to summarize the occupational burden into one variable.

**Methods:**

A population-based case–control study conducted in Germany in 1998–2000 included 208 male cases and 702 controls. Information on occupational history, smoking, alcohol consumption and education was collected with face-to-face interviews. A recently developed job-classification index was used to account for the occupational burden. A sub-index focussed on jobs involving potentially carcinogenic agents (CAI) for the upper aero digestive tract.

**Results:**

When adjusted for smoking and alcohol consumption, higher odds ratios (ORs) were found for lower education. This OR decreased after further adjustment using the physical and psycho-social job indices (OR = 3.2, 95%-CI: 1.5-6.8), similar to the OR using the sub-index CAI (OR = 3.0, 95%-CI: 1.4-6.5).

**Conclusions:**

The use of an easily applicable control variable, simply constructed on standard occupational job classifications, provides the possibility to differentiate between educational and occupational contributions. Such an index might indirectly reflect the effect of carcinogenic agents, which are not collected in many studies.

## Background

Laryngeal cancer is all over the world more common in males than in females. The age-standardized incidence rates (ASR) in 2008 for Europe were 9.5 and 0.8 per 100,000 for males and females respectively with somewhat lower mortality rates (5.0 and 0.3), showing lower rates in Northern than Southern Europe. In Germany the ASRs in 2008 for males and females were 7.2 and 0.8 per 100,000, respectively, with their according mortality rates of 2.4 and 0.3 per 100,000 [1]. The relationship between social inequalities and laryngeal cancer has been mentioned over the recent years by various authors: Conway and colleagues found a significant association between a low socioeconomic status (SES) and oral cancer risk [2] and two other studies quantitatively assessed the proportions attributable to lifestyle and occupational exposure using various socioeconomic indicators such as education, occupational class and income [3-5]. It has been estimated that 70% to 85% of laryngeal cancers are caused by smoking and 25% by alcohol [4,6], with an estimate for the combined effect of about 89% [7]. As major risk factors, tobacco and alcohol consumption should be considered first when investigating the mechanisms leading to socioeconomic inequalities in laryngeal cancer incidence. However, they alone do not totally explain the observed social inequalities. Occupational exposure to carcinogenic agents may account for a part of the residual effects [3]. Indeed, exposure to coal dust, asbestos, cement, wood and hard-alloys dust, chlorinated solvents, polycyclic aromatic hydrocarbons were found to be associated with laryngeal cancer risk [8-12]. Obviously, those exposures are more prevalent in lower socioeconomic classes due to the close relation of SES and occupational possibilities. Additional factors mentioned in the aetiology of laryngeal cancer or head and neck cancer in general are environmental exposures, physical activity, physical and psychological stress and social aspects [13,14]. However, these seem to play a minor role than occupational factors.

In occupational cohort studies, job exposure matrices (JEMs) are accepted tools for assessing occupational exposures [15]. Traditionally, such exposure matrices have been developed for chemical exposures, but only few of them include estimates of physical or psycho-social workload factors [16-18]. Moreover, JEMs are not available for all jobs mentioned in the International Standard Classification of Occupation (ISCO). Recently, a German working group constructed a set of occupational indices based on JEMs to circumvent this problem and link all the available ISCO-classified jobs to an index value [19]. The aim of this study was to evaluate the contribution of occupation using occupational indices differentiating between physical, psycho-social and toxic exposures summarizing the occupational burden in one variable. These previously validated indices which use a survey based classification of job demands [19] are simple to apply to occupational history data and might be able to explain a large part of the socioeconomic differences in laryngeal cancer.

## Methods

This population-based case–control study was conducted in Germany with 208 histological confirmed male cases (response rate 9.2%) between 1998 and 2000. The study region covered a population of about 2.7 million people in South-West Germany, comprising the cities of Heidelberg, Mannheim, Ludwigshafen, Darmstadt, and Heilbronn. Cases and controls were restricted to Germans aged ≤80 who were registered as citizens in the study region. 702 population controls were selected randomly from the population registries of the study area and were originally 1:3 frequency-matched for age (response rate 62.4%). After checking the clinical-pathological records, 28 patients had to be excluded due to other diagnoses or recurrence of an earlier tumour. Ethical clearance was received by the ethical committee of the University of Heidelberg and written consent was obtained from the participants through collaborating physicians.

Risk factors were obtained with face-to-face interviews using a detailed standardized questionnaire [20]. Information on smoking, alcohol consumption and occupational exposure was collected with a comprehensive, standardized questionnaire which has been used in almost identical form in previous large studies [21,22].

SES was assessed in terms of education and grouped in three levels according to the years of school attended following the German educational system: nine years or less (“Hauptschule”), 10 years (“mittlere Reife”) and more than 10 years (“(Fach-)Hochschulreife”).

Smoking data were assessed by lifetime smoking periods for which daily, weekly and monthly tobacco consumption of cigarettes (rare uses of cigars, cigarillos and pipes were added according to their average weight relative to that of cigarettes) and were used to calculate pack-years of smoking, i.e. the cumulative number of cigarette smoked (1 pack-year corresponds to 20 cigarettes/day for one year, being equivalent to nearly 7300 cigarettes). Pack-years were included as a log-transformed continuous variable, which showed statistically the best model fit using the fractional polynomial technique [23]. Time since smoking cessation was included as binary variable “having stopped smoking at least 2 years before diagnosis/interview”.

Alcohol consumption was calculated from the daily, weekly or monthly consumption 10 years before interview for all common alcoholic beverages, assuming the following ethanol content: beer 5%, wine, fruit wine or sparkling wine 10%, aperitif and liquors 20% and spirits 40%. Average daily consumption was included as an untransformed continuous variable, again following the fractional polynomial technique. More detailed information about the study population and the assessment of smoking and alcohol consumption can be found elsewhere [6].

A detailed life-time occupational history section collected data on every occupation since the time point people started their working phase, including start date, end date, job title, industry and nature of work. More details about the assessment of the occupational history can be found elsewhere [8,9,11,12]. Each job title was coded according to the International Standard Classifications for Occupation (ISCO) and converted from ISCO-68 to ISCO-88 [24] as the latter one served as a basis for the application of the previously published occupational indices used in our study here [19].

As the occupational indices used in this paper were previously only published in German language, some construction details for the indices will be given here for a better understanding. In the work done by Kroll and colleagues, the occupational burden is measured via JEMs that were constructed specifically for Germany and matched to data using the International Classification of Occupations of 1988 (ISCO-88) by the International Labour Organization [25]. In these JEMs, 100% of all ISCO-88 2-digit codes, 94.8% of the 3-digit codes and 78.5% of the 4-digit codes are represented. The JEMs were based on data from a large scale representative survey on working conditions for 20.000 employees in Germany. The German survey was based on the European Working Conditions Survey conducted regularly since the 1980s in all Member States of the European Union [26] on demand of the European Commission [26,27]. The JEMs were constructed using hierarchical linear regression models (HLM) using summary scores for job exposures in three domains based on 39 individual job characteristics [19]. The levels for the multi-level estimation were defined by the 4-digit codes of the ISCO-88 classification and the respondents of the survey. 5 dimensions of occupational burden were analysed: Ergonomic Stress (ES), Environmental Pollution (EP), Mental Stress (MS), Social Stress (SS) and Temporal Loads (TL). The individual scores for the items were summed up for each dimension to build the indices.

The Overall Job Index (OJI) is defined as the sum of all these dimensions. A Physical Job Index (PJI) was constructed using ES and EP only, whereas a Psycho-Social Index (PSI) includes MS, SS and TL. An additional index considering only those jobs with a likely exposure to smoke, dust, gases and vapors was summarized as Carcinogenic Agent Index (CAI). Thus, the items of the CAI are a subset of the items of the PJI, as the PJI is a subset of the OJI. The indices are controlled for respondent characteristics such as age, gender, working hours and experience on the job. They were validated externally using data of the German Health Update 2009 [19] and the German Socio-Economic Panel Study [28]. The indices’ values refer to deciles of jobs according to the conducted German survey in ascending order: jobs in index group 1 were among those with the lowest occupational burden (like draftsmen, bookkeepers and teachers), index group 10 refers to a particularly heavily loaded group (like miners, bricklayers and metal and machinery workers) in comparison to all occupational groups. In the original concept, the indices were categorized in three levels [19]: “high” (index values 9–10), “middle” (index values 3–8) and “low” (index values 1–2). Due to the broad interval of deciles included in the middle level of the job indices, we divided it into “upper-middle” (6–8) and “lower middle” (3–5).

In our study we matched the indices both with all jobs mentioned in the lifetime job history and with the longest job. For the analyses, the indices were used as ordinal and categorical variables.

To illustrate which exposures might play a role in jobs which are associated to a high value of CAI, we linked exposure information independently collected through a substance check list (SCL) via year of exposure and year of job period: we summarized the working hours of cases and controls respectively per year and substance and linked them to the CAI via year of exposure and year of job period. Since in the same year a person could potentially have been exposed to different substances, parallel exposures to multiple substances reported in the SCL during the same year were registered. To account for different intensities, we used the reported hours of exposure per substance by cases and controls as exposure unit.

Odds Ratios (OR) and 95%-Confidence Intervals (CI) were assessed by conditional logistic regression models conditioned on age (five years age groups) [29]. The models were adjusted for smoking cessation, tobacco and alcohol consumption and the occupational indices. The statistical software package SAS (version 9.2) was used for the analysis.

## Results

**Figure 1:**
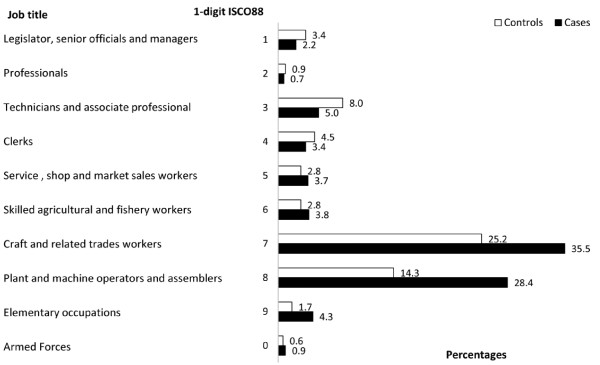
**Distribution of worked years by cases and controls with less than 10 years of school.** Percentages are calculated on the total years worked by cases and controls (8440.5 and 28894 respectively).

Two hundred eight cases and 702 controls participated in this study. Results on the basic characteristics like the age distribution and smoking and alcohol behaviour can be found elsewhere [6]. The average age of participants was 63 years, with a mean number of 3.6 jobs and 39 years worked for cases, similar to a mean of 3.3 jobs and 40 years worked for controls. The majority of cases (87%) attended school for less than 10 years. Thus, results shown here are mainly based on this educational category, due to small numbers in the higher educational groups. In their lifetime occupational history, a majority of cases (63.9%) with less than 10 years of school worked as constructors, machine operators and assemblers (1-digit ISCO-88: 7 and 8) in comparison to 39.5% controls (Figure 1). These percentages are calculated on a total of 8440.5 worked years by cases and 28894 worked years by controls, reflecting nearly perfectly the matching ratio.

**Figure 2:**
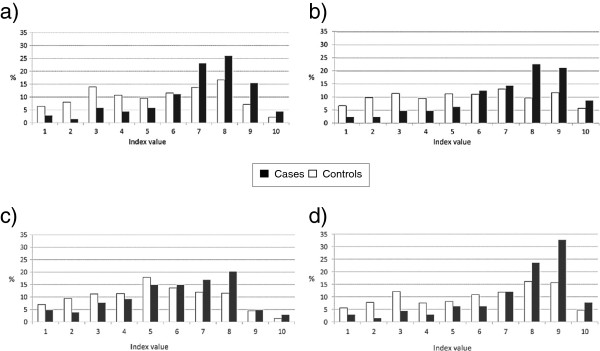
Distribution of (a) Overall Job Index (OJI), (b) Physical Job Index (PJI), (c) Psycho-Social Job Index (PSI), (d) Carcinogenic Agent Index (CAI) in cases and controls.

The application of the indices to data of our case–control study shows significantly different distributions and medians for all indices, with higher levels for cases in comparison to controls, most pronounced in CAI (Figure 2). The mean scores for cases and controls respectively were 7.2 versus 5.8 (OJI), 7.4 versus 5.8 (PJI) and 7.8 versus 6.3 (CAI). Interestingly cases resulted also to have a higher psycho-social burden (6.2) than controls (5.4) (Figure 2c).

As all jobs performed during lifetime were linked to the occupational indices, a person could appear more than once in different index categories (see Table 1 (OJI) and Table 2 (CAI)). For example, a person who worked for 12 years as a bricklayer (OJI-index value: 10) and for 9 years as a building electrician (OJI-index value: 8) contributes to two different categories of the index. The percentage of years worked in jobs with the highest OJI was nearly doubled in cases (36.0%) than in controls (19.8%) and vice versa in jobs with low OJI (6.1% versus 14.9%, respectively). This “high” OJI group presents mainly jobs like bricklayers, carpenters, miners, mechanics, metal and machinery workers (ISCO-88 groups: 71, 72, 82 and 83) (Table 1). Also the “upper middle” category of the OJI i.e. referring to the index values 6, 7 and 8 showed a higher percentage of lifelong years worked by cases (45.7%) than controls (36.8%). This index group is dominated by jobs from the ISCO-88 groups 81, 82, 72 and 83 (like mining and chemical plant operators, well drillers and borers; mechanical, metal products assemblers, agricultural or industrial machinery mechanics and fitters).

**Table 1:** Distribution of job years worked for cases and controls according to ISCO-88 in levels of the Overall Job Index (OJI)

**Description of jobs (ISCO88-2 digits)**	**Cases%**	**Controls%**
**High**	**36.0**	**19.8**
Bricklayers, plumbers, carpenters, miners, building constructors, roofers (71)	15.1	8.4
Mechanics, metal and machinery workers (72)	7.7	5.1
Printing-, plastic-, wood-, textile-, chemical-products machine operators (82)	4.8	1.9
Agricultural and earth-moving plant operators (83)	3.5	0.8
Forestry workers (61)	1.8	1.9
Fire fighters, cooks and waiters (51)	1.2	0.9
Others ^a^	1.9	0.8
**Upper middle**	**45.7**	**36.8**
Mining, chemical- processing plant operators, well drillers and borers, paper-pulp and chemical-heat treating plant operators (81)	9.1	5.1
Mechanical, metal products assemblers (82)	5.9	3.2
Agricultural or industrial machinery mechanics and fitters, electrical and electronic equipment mechanics and fitters (72)	5.5	5.4
Heavy-truck drivers (83)	4.7	4.3
Building electricians (71)	3.2	2.1
Printing engravers and etchers, glass and ceramic decorators (73)	2.5	1.4
Cabinet makers (74)	2.6	3.6
Market gardeners (61)	2.0	1.5
Mail and sorting clerks (41)	1.8	3.7
Shop salespersons and demonstrators (52)	1.6	1.6
Police officers (51)	1.4	0.8
Agricultural and fishery workers (92)	1.3	0.4
General managers in wholesales, restaurants and hotels (13)	1.0	1.5
Others ^a^	3.1	2.2
**Lower middle**	**12.4**	**28.5**
Directors, production and operations department managers (12)	3.1	6.6
Buyers, trade brokers, business services agents and trade brokers, athletes, sportspersons, police inspectors and detectives, decorators and commercial designers (34)	2.3	6.1
Chemical, physical science, civil, electronics and telecommunications engineering, mechanical technicians, computer assistants (31)	1.3	4.2
Car, taxi and van drivers (83)	1.3	0.7
Computer system designers, civil engineers, architects (21)	1.1	2.3
Authors, journalists and other writers (24)	0.9	0.5
Teachers (23)	0.4	2.8
Others ^a^	2.0	5.3
**Low**	**6.1**	**14.9**
Accounting and book-keeping clerks (41)	2.8	5.1
Bookkeepers, administrative secretaries (34)	2.0	3.8
Mechanical, electrical, electronics and telecommunications engineers, mathematicians (21)	0.9	0.6
Business professionals, judges, interpreters, psychologists, religious professionals (24)	0.4	1.9
Others ^a^	0.0	3.5

^a^job categories with a frequency of less than 1%.

Total percentage of job years for each category of OJI are presented in bold.

**Table 2:** Distribution of job years worked for cases and controls according to ISCO-88 in levels of the Carcinogenic Agent Index (CAI)

**Description of jobs (ISCO88-2 digits)**	**Cases %**	**Controls %**
**High**	**55.6**	**33.9**
Building electricians, bricklayers and miners, roofers (71)	18.3	10.5
Metal and machinery workers (72)	10.6	5.2
Chemical- and wood- processing and power- production plant operators (81)	10.4	5.1
Printing-, plastic-, wood-, rubber-, cement-, textile- and chemical- products machine operators (82)	7.1	3.0
Agricultural and earth-moving plant operators (83)	2.9	0.4
Bakers, cabinet makers (74)	2.5	3.3
Forestry workers (61)	1.8	1.8
Others ^a^	2.0	4.6
**Upper middle**	**27.0**	**29.7**
Heavy-truck, car, taxi, van, bus, tram drivers (83)	6.2	5.3
Mechanical machine assemblers (82)	3.6	2.0
Machine-tools, electrical equipment setters and fitters (72)	2.8	4.0
Compositors, typesetters, printing engravers and etchers (73)	2.3	1.2
Market gardeners, crop- and tree growers, dairy and livestock producers (61)	2.1	1.5
Cooks and police officers (51)	1.9	1.5
Shops sales persons and demonstrators (52)	1.6	1.6
Physical science, mechanical, physical, electrical and electronics engineering technicians, quality inspectors (31)	1.3	4.6
Street vendors, building caretakers (91)	1.2	0.7
Agricultural, fishery and related workers (92)	1.0	0.2
General managers in wholesales, restaurants and hotels (13)	1.0	1.6
Others ^a^	2.0	5.5
**Lower middle**	**12.0**	**22.9**
Data entry, stock, transport, mail and sorting clerks (41)	3.3	4.5
Directors, production and operations department managers (12)	2.6	4.2
Customs, tax professionals, technical and commercial sales representatives, social work associate professionals (34)	1.9	4.3
Draughts persons, civil engineering technicians, broadcasting and telecommunications equipment operators (31)	1.3	1.4
Civil engineers, architects (21)	0.4	4.2
Others ^a^	2.5	4.3
**Low**	**5.5**	**13.6**
Insurance representative, estate agents, buyers, bookkeepers (34)	2.4	5.5
Business professionals, judges, interpreters, psychologists, religious professionals, legal professionals, writers, journalists, economists (24)	1.4	2.3
General department managers (12)	0.4	1.2
College, university and higher education teachers (23)	0.0	0.9
Others ^a^	1.3	3.7

^a^job categories with a frequency of less than 1%.

Total percentage of job years for each category of CAI are presented in bold.

The job distribution was similar, but much more pronounced in the CAI. Here, 55.6% of cases worked in jobs with a high level of CAI in contrast to 33.9% of controls (Table 2). Building electricians, agricultural or industrial machinery mechanics, machine tool operators and chemical-, wood-processing and power-production plant operators account for additional 19.6% of years worked for cases and 14.1% for controls compared to the “high” category of the OJI.

**Figure 3:**
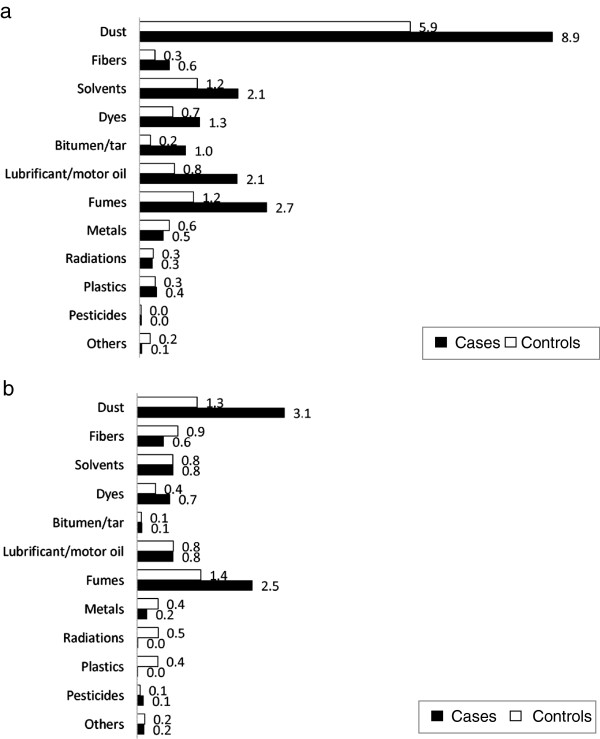
Lifetime Hours of Exposure (in 1.000) per person to reported Substances (SCL) in High (index value 9–10) (a) and Upper-middle (index value 6–8) (b) categories of the Carcinogenic Agent Index (CAI).

One hundred and eighty five cases (89%) and 542 controls (77%) reported an exposure to the provided substance check list. After linkage of the jobs and their corresponding CAI to the reported substances in the SCL of each individual, participants represented in the category “high” were mainly exposed to dust, with 8926 lifetime exposure hours per case in contrast to 5854 lifetime exposure hours per control (Figure 3a). Within this category of dust exposure, cases were more in contact with metal dust (3239 lifetime hours versus 385 hours for controls), stone and sand dust (1458 lifetime hours versus 907 for controls), cement (1311 lifetime hours versus 867 for controls) and soft- and hardwood (1614 lifetime hours versus 826 for controls) (data not shown). Cases showed higher exposure hours per person for fumes, solvents and oils than controls. The same pattern was given with a high ratio between cases and controls, but a lower number of exposure hours for bitumen (ratio 5:1), dyes and fibres. Again, a similar pattern could be seen in the upper middle category of CAI (Figure 3b).

Stratifying for age only, laryngeal cancer was strongly associated with a lower educational level (crude OR: 6.0; 95% CI: 3.1-11.4) (Table 3). After adjustment for smoking cessation, tobacco and alcohol consumption, significant ORs could be seen in all models using the different occupational indices. These ORs were all in the same order of magnitude where the best model fit was given by a model containing CAI. Here the OR for less than 10 years of school vs. more than 10 years was 3.0 (95% CI: 1.4-6.5). Models including interaction terms between occupation/smoking, occupation/alcohol or occupation/education did not show any significant interaction.

**Table 3:** Distribution and Odds ratios for education in models including smoking, alcohol consumption and occupational indices

	**Cases**	**Controls**	**Univariate**	**Adjusted for smoking**^**b **^**and alcohol**^**c**^	**Adjusted for smoking**^**b **^**and alcohol**^**c**^	**Adjusted for smoking**^**b **^**and alcohol**^**c**^	**Adjusted for smoking**^**b **^**and alcohol**^**c**^
					**+ OJI**^**d**^	**+ PJI + PSI**^**d**^	**+ CAI**^**d**^
	**N (%)**	**N (%)**	**OR**^**a**^**(95% CI)**	**OR**^**a**^**(95% CI)**	**OR**^**a**^**(95% CI)**	**OR**^**a**^**(95% CI)**	**OR**^**a**^**(95% CI)**
**Years of school**							
More than 10 years	11 (5.3)	161 (22.9)	1.0	1.0	1.0	1.0	1.0
10 years	16 (7.7)	105 (15.0)	2.2 (0.99-5.0)	1.7 (0.7-3.9)	1.5 (0.6- 3.6)	1.5 (0.6- 3.7)	1.5 (0.6- 3.6)
Less than 10 years	181 (87.0)	436 (62.1)	6.0 (3.1- 11.4)	4.7 (2.4-9.3)	3.2 (1.5- 6.7)	3.2 (1.5- 6.8)	3.0 (1.4-6.5)

^a^OR, odds ratio; CI, confidence interval. All models were stratified by age.

^b^Smoking variables: quitting smoking, smoking status in pack-years (0, <20, 20–40, >40).

^c^Alcohol variables: alcohol consumption in grams ethanol/day (<25, 25–75, >75).

^d^OJI, Overall Job Demand; PJI, Physical Job Demand; PSI, Psycho-social job demand; CAI, Carcinogenic Agent Exposure. The average of all indices per person for all lifetime performed jobs; per 2 units increase of the respective indices.

Models estimating the contribution of the different occupational indices based on the longest job also showed a significant association: in the model including CAI, the adjusted OR for the lowest educational level was 3.3 (95% CI: 1.6-7.1), similar to the result for the lifetime occupation (data not shown).

## Discussion

The aim of the present study was to show that occupational aspects play a main role in the contribution of education as a risk factor for laryngeal cancer. We applied several job-related indices linked via job titles using ISCO-88 to distinguish between the educational inequalities in laryngeal cancer risk and the occupational burden. We showed that the exposure of a range of jobs can be aggregated into a single variable, starting from an overall job demand index that can differentiate between physical, psycho-social and carcinogenic aspects of occupational burden.

The overall index (OJI) combines 5 dimensions of the occupational burden (ergonomic stress, environmental pollution, mental stress, social stress and temporal loads) into one index. Although this OJI does not differentiate between the individual aspects, its inclusion reduces the Odds Ratio for low education from 4.7 (95% CI: 2.4-9.3) to 3.2 (95% CI: 1.5-6.7) after adjusting for smoking and alcohol consumption. The OR for education does not decrease further when this overall index is split into physical (PJI) and psycho-social (PSI) dimensions. The distributions of all indices and their medians were significantly different between cases and controls. However, the psycho-social job dimension plays only a small role in our study, in agreement with the existing literature on laryngeal cancer [30,31]. The physical burden and especially the inhalative exposure to carcinogenic substances condensed in the carcinogenic agent index play a much more important role. Nevertheless, the psycho-social aspect was worth to consider, as previous studies asked to deepen this aspect in laryngeal cancer as well [32].

The PJI is used as an overall assessment for physical and environmental demands while the CAI, as a subset of the OJI, is designed to focus just on carcinogens. Nonetheless both indices are highly correlated on the ISCO-88 4 digit level (r = .87), while the correlation with the OJI is a bit smaller (r = .79) (data not shown). This means, exposure to smoke, dust, gases and vapors is very frequent in many jobs with high physical demands. This CAI showed the strongest decrease in the OR for the educational variable.

The indices are easily applied in any case where job information is available. As clinical data often only collects the longest job recalled by the patient, the same analysis using the longest job only were conducted and showed significant associations of low education with laryngeal cancer for the different index dimensions (data not shown).

The extracted sub- index (CAI) showed the potential to indirectly reflect the burden of known or suspected carcinogens for laryngeal cancer, after linkage to all exposures reported in a separate substance checklist. This is especially important, as usually occupational studies or clinical records collect job titles, but do not collect exposure details to carcinogenic agents. The group with the highest CAI was mainly exposed to dust, fumes and solvents, replicating previous results from the same study which showed that wood dust, polycyclic aromatic hydrocarbons and cement dust are independent risk factors for laryngeal cancer [8,9,11,12]. Additionally, the CAI identifies jobs with high occupational burden, such as bricklayers and building construction workers, which were already found to have a high risk for laryngeal cancer in the recent ARCAGE study [33].

However, the use of an index which reflects better working conditions in 2006 compared to those in the 1960s or 1970s might underestimate the real occupational burden. Employment pollution was surely higher in previous years, where programs for occupational health and safety were at most in the initial phase. Thus, we conducted some sensitivity analyses, changing the CAI levels for those jobs where we assumed a higher burden in the past (like industrial workers and painters). This resulted in a higher reduction of the OR for education. An underestimation might be given for alcohol consumption as well, as patients tend to underreport exposures. In a sensitivity analysis, we applied a second independent alcohol variable with a lower mean reported consumption, resulting in a lower contribution of alcohol consumption. The same might hold for smoking. SES was measured through educational level that allows classification of all individuals, regardless of age and working circumstances. It usually predicts, and to some degree determines, employment and the ability to earn income and consequently access the health care system [34]. Although underrepresentation of controls in the lowest educational level cannot be ruled out, this could not be proven as educational information for the 40ties and 50ties were not available because census data were collected starting from year 1975 [35]. Median age in our study was 63 years. Thus, around 50% of the study population was affected by the educational situation during or after World War 2nd. Our data showed a trend in the risk of laryngeal cancer through all educational levels. However, no significant ORs were found for the middle level of education. This category includes people who attended an apprenticeship in a vocational school (“Berufsschule”) having the chance for career development up to low management level, representing a probable lower occupational burden.

Among other sources of bias in case–control studies, low response rates in controls may lead to a non-representative sample of controls and to confounding/residual confounding due to missing or insufficient control for other risk factors [36]. Our response rate was 62.4%, which in our view is very satisfactory in comparison to other studies. However, It does not rule out a possible bias. The response rates are lower for the youngest and for the oldest age groups. Unfortunately, no information on non-responders is available for this study. As both smoking and excessive alcohol consumption are strong risk factors for laryngeal cancer we did a careful investigation on residual confounding. The confounding effect, in particular of smoking, was clearly visible. Most OR estimates reduced toward one after adjustment. We also checked for different methods of adjustment, using smoking/alcohol as categorical variable. Differences, however, were negligible for different methods of adjustment.

The significant association between the lowest educational level and laryngeal cancer and its decreasing after adjustment for tobacco and alcohol consumption found in our study is in line with previous findings [4,5]. Although the magnitude of the association was similar between our results and these previous ones, a direct comparison is not possible as the level of education was recorded in different ways taking into account the education system of the country under study. However, some authors derived the job classification and the occupational exposure index from the same variable; therefore, they are inherently correlated [3]. Boing et al. [5] tried to account for the collinearity between education and occupation fitting separate models, but as there are structural links among education, income and occupational class all three would give rise to a similar distribution of any specific outcome. Moreover, a dichotomous classification of job (manual versus non-manual) correlates with many social indicators such as income, health, and educational attainment, as well as conditions of employment broadly defined and therefore less informative [37]. We checked different models considering pairwise interaction terms between our educational and occupational variables, as well as smoking behaviour and alcohol consumption. However, no significant interaction between these variables were found.

## Conclusions

In conclusion, with the use of an easily applied control variable, simply constructed using standard occupational job classifications, we were able to differentiate the effects of occupational components, such as physical and psycho-social demand on laryngeal cancer risk. The occupational indices summarize occupational information for all ISCO-coded jobs, as valid job exposure matrices for many industrial sectors are not available and would not cover the range of 178 jobs (on a 3-digit ISCO-68 basis) considered in our study. Much more important is the fact that such an index can indirectly reflect the effect of carcinogenic agents, which are often not directly collected in case–control studies. Thus, we suggest using these occupational indices as additional adjustment variables.

## Competing interests

The authors declare that they have no conflict of interests.

## Authors’ contributions

IS conducted the data analyses and drafted the manuscript. LEK provided the occupational index and contributed to the methodological part of the manuscript. AD, HB and HR conducted the study, where HR led the present project. All authors participated in writing the manuscript and read and approved the final version.

## Pre-publication history

The pre-publication history for this paper can be accessed here:

http://www.biomedcentral.com/1471-2458/13/1080/prepub

## References

[B1] Ferlay J Parkin DM Steliarova-Foucher E Estimates of cancer incidence and mortality in Europe in 2008 Eur J Cancer 2010 46 4 765 781 Available from: http://dx.doi.org/10.1016/j.ejca.2009.12.014 10.1016/j.ejca.2009.12.014 20116997

[B2] Conway DI Petticrew M Marlborough H Berthiller J Hashibe M Macpherson LMD Socioeconomic inequalities and oral cancer risk: a systematic review and meta-analysis of case–control studies Int J Cancer 2008 122 12 2811 2819 Available from: http://dx.doi.org/10.1002/ijc.23430 10.1002/ijc.23430 18351646

[B3] Menvielle G Luce D Goldberg P Leclerc A Smoking, alcohol drinking, occupational exposures and social inequalities in hypopharyngeal and laryngeal cancer Int J Epidemiol 2004 33 4 799 806 Available from: http://dx.doi.org/10.1093/ije/dyh090 10.1093/ije/dyh090 15155704

[B4] Conway DI McKinney PA McMahon AD Ahrens W Schmeisser N Benhamou S Socioeconomic factors associated with risk of upper aerodigestive tract cancer in Europe Eur J Cancer 2010 46 3 588 598 Available from: http://dx.doi.org/10.1016/j.ejca.2009.09.028 10.1016/j.ejca.2009.09.028 19857956

[B5] Boing AF Antunes JLF de Carvalho MB de Góis Filho JF Kowalski LP Michaluart P Jr How much do smoking and alcohol consumption explain socioeconomic inequalities in head and neck cancer risk? J Epidemiol Community Health 2011 65 8 709 714 Available from: http://dx.doi.org/10.1136/jech.2009.097691 10.1136/jech.2009.097691 20724282

[B6] Ramroth H Dietz A Becher H Interaction effects and population-attributable risks for smoking and alcohol on laryngeal cancer and its subsites. A case–control study from Germany Methods Inf Med 2004 43 5 499 504 Available from: http://dx.doi.org/10.1267/METH04050499 15702209

[B7] Hashibe M Brennan P Chuang SC Boccia S Castellsague X Chen C Interaction between tobacco and alcohol use and the risk of head and neck cancer: pooled analysis in the International Head and Neck Cancer Epidemiology Consortium Cancer Epidemiol Biomarkers Prev 2009 18 541 550 Available from: http://dx.doi.org/10.1158/1055-9965.EPI-08-0347 10.1158/1055-9965.EPI-08-0347 19190158 PMC3051410

[B8] Dietz A Ramroth H Urban T Ahrens W Becher H Exposure to cement dust, related occupational groups and laryngeal cancer risk: results of a population based case–control study Int J Cancer 2004 108 6 907 911 Available from: http://dx.doi.org/10.1002/ijc.11658 10.1002/ijc.11658 14712496

[B9] Becher H Ramroth H Ahrens W Risch A Schmezer P Dietz A Occupation, exposure to polycyclic aromatic hydrocarbons and laryngeal cancer risk Int J Cancer 2005 116 3 451 457 Available from: http://dx.doi.org/10.1002/ijc.21049 10.1002/ijc.21049 15810012

[B10] Shangina O Brennan P Szeszenia-Dabrowska N Mates D Fabiánová E Fletcher T Occupational exposure and laryngeal and hypopharyngeal cancer risk in central and eastern Europe Am J Epidemiol 2006 164 4 367 375 Available from: http://dx.doi.org/10.1093/aje/kwj208 10.1093/aje/kwj208 16801374

[B11] Ramroth H Dietz A Ahrens W Becher H Occupational wood dust exposure and the risk of laryngeal cancer: a population based case–control study in Germany Am J Ind Med 2008 51 9 648 655 Available from: http://dx.doi.org/10.1002/ajim.20605 10.1002/ajim.20605 18626911

[B12] Ramroth H Ahrens W Dietz A Becher H Occupational asbestos exposure as a risk factor for laryngeal carcinoma in a population-based case–control study from Germany Am J Ind Med 2011 54 7 510 514 Available from: http://dx.doi.org/10.1002/ajim.20963 10.1002/ajim.20963 21538446

[B13] Marmot M Social determinants of health inequalities Lancet 2005 365 9464 1099 1104 Available from: http://dx.doi.org/10.1016/S0140-6736(05)71146-6 15781105 10.1016/S0140-6736(05)71146-6

[B14] Marmot M Wilkinson RG Psychosocial and material pathways in the relation between income and health: a response to Lynch et al BMJ 2001 322 7296 1233 1236 10.1136/bmj.322.7296.1233 11358781 PMC1120336

[B15] Leclerc A Exposure assessment in ergonomic epidemiology: is there something specific to the assessment of biomechanical exposures? Occup Environ Med 2005 62 3 143 144 Available from: http://dx.doi.org/10.1136/oem.2004.017889 10.1136/oem.2004.017889 15723877 PMC1740982

[B16] Seidler A Bolm-Audorff U Heiskel H Henkel N Roth-Küver B Kaiser U The role of cumulative physical work load in lumbar spine disease: risk factors for lumbar osteochondrosis and spondylosis associated with chronic complaints Occup Environ Med 2001 58 11 735 746 10.1136/oem.58.11.735 11600730 PMC1740072

[B17] Kauppinen T Toikkanen J Pukkala E From cross-tabulations to multipurpose exposure information systems: a new job-exposure matrix Am J Ind Med 1998 33 4 409 417 10.1002/(SICI)1097-0274(199804)33:4<409::AID-AJIM12>3.0.CO;2-2 9513649

[B18] Cohidon C Santin G Chastang JF Imbernon E Niedhammer I Psychosocial exposures at work and mental health: potential utility of a job-exposure matrix J Occup Environ Med 2012 54 2 184 191 Available from: http://dx.doi.org/10.1097/JOM.0b013e31823fdf3b 10.1097/JOM.0b013e31823fdf3b 22249578

[B19] Kroll L Construction and Validation of a General Index for Job Demands in Occupations Based on ISCO-88 and KldB-92 Methoden — Daten — Analysen 2011 Jg. 5, Heft 1 63 90

[B20] Ramroth H Altenburg HP Becher H Auswahl von Populationskontrollen für epidemiologische Fall-Kontroll-Studien unter Verwendung regionaler Stichproben Informatik Biometrie und Epidemiologie in Medizin und Biologie 2001 32 1 60

[B21] Ahrens W Jöckel KH Brochard P Bolm-Audorff U Grossgarten K Iwatsubo Y Retrospective assessment of asbestos exposure–I. Case–control analysis in a study of lung cancer: efficiency of job-specific questionnaires and job exposure matrices Int J Epidemiol 1993 22 Suppl 2 S83 S95 8132398 10.1093/ije/22.supplement_2.s83

[B22] Harris MA Cripton PA Teschke K Retrospective assessment of occupational exposure in case–control studies J Occup Environ Hyg 2012 9 6 371 380 10.1080/15459624.2012.679853 22571854

[B23] Royston P Ambler G Sauerbrei W The use of fractional polynomials to model continuous risk variables in epidemiology Int J Epidemiol 1999 28 5 964 974 10.1093/ije/28.5.964 10597998

[B24] Ganzeboom HBG Treiman DJ “International Stratification and Mobility File: Conversion Tools” 2009 http://home.fsw.vu.nl/hbg.ganzeboom/ismf

[B25] Organization IL International standard Classification of Occupations - MAJOR, SUB-MAJOR, MINOR AND UNIT GROUP TITLES 2004 Available from: http://www.ilo.org/public/english/bureau/stat/isco/isco88/major.htm

[B26] Hartmann J BIBB/BAuA-Erwerbstätigenbefragung 2005/2006 Feldbericht 2006 München: TNS Infratest Sozialforschung

[B27] Parent-Thirion A HJea Macias EF Fourth European Working Conditions Survey 2007 Luxembourg: Office for Official Publications of the European Communities

[B28] Kroh M Neiss H Kroll L Lampert T Menschen mit hohem Einkommen leben länger. [High income people live longer] DIW-Wochenbericht 2012 33 3 15

[B29] Neuhäuser M Becher H Improved odds ratio estimation by post hoc stratification of case–control data Stat Med 1997 16 9 993 1004 10.1002/(SICI)1097-0258(19970515)16:9<993::AID-SIM505>3.0.CO;2-2 9160494

[B30] van Loon AJ Tijhuis M Surtees PG Ormel J Lifestyle risk factors for cancer: the relationship with psychosocial work environment Int J Epidemiol 2000 29 5 785 792 10.1093/ije/29.5.785 11034957

[B31] Sampson W Controversies in cancer and the mind: effects of psychosocial support Semin Oncol 2002 29 6 595 600 Available from: http://dx.doi.org/10.1053/sonc.2002.50008 10.1053/sonc.2002.50008 12516043

[B32] Ahrens W Jöckel KH Patzak W Elsner G Alcohol, smoking, and occupational factors in cancer of the larynx: a case–control study Am J Ind Med 1991 20 4 477 493 10.1002/ajim.4700200404 1785612

[B33] Richiardi L Corbin M Marron M Ahrens W Pohlabeln H Lagiou P Occupation and risk of upper aerodigestive tract cancer: the ARCAGE study Int J Cancer 2011 Available from: http://dx.doi.org/10.1002/ijc.26237 10.1002/ijc.26237 21671472

[B34] Galobardes B Shaw M Lawlor DA Lynch JW Davey Smith G Indicators of socioeconomic position (part 1) J Epidemiol Community Health 2006 60 1 7 12 Available from: http://dx.doi.org/10.1136/jech.2004.023531 10.1136/jech.2004.023531 16361448 PMC2465546

[B35] Landesamt Baden-Württemberg S. Abgänger aus öffentlichen und privaten allgemeinbildenden und beruflichen Schulen in Baden-Württemberg 1975 bis 2010 nach Abschlussart und Schulart Available from: http://www.statistik.baden-wuerttemberg.de/BildungKultur/Landesdaten/

[B36] Geneletti S Richardson S Best N Adjusting for selection bias in retrospective, case–control studies Biostatistics 2009 10 1 17 31 Available from: http://dx.doi.org/10.1093/biostatistics/kxn010 18482997 10.1093/biostatistics/kxn010

[B37] Marshall G Manual versus non-manual distinction A Dictionary of Sociology 1998 *Encyclopedia.com.* 22 Nov. 2013 http://www.encyclopedia.com

